# A systematic review of techniques and interventions for improving adherence to inclusion and exclusion criteria during enrolment into randomised controlled trials

**DOI:** 10.1186/1745-6215-11-17

**Published:** 2010-02-23

**Authors:** Fiona Simpson, Elizabeth A Sweetman, Gordon S Doig

**Affiliations:** 1 Department of Intensive Care, Northern Clinical School, University of Sydney, Sydney, NSW, Australia; 2 Intensive Care Unit, Department of Intensive Care, Royal North Shore Hospital, Pacific Highway, St. Leonards, NSW 2065, Australia

## Abstract

**Background:**

Enrolment of patients into a randomised controlled trial (RCT) in violation of key inclusion or exclusion criteria, may lead to excess avoidable harm. The purpose of this paper was to systematically identify and review techniques and interventions proven to prevent or avoid inappropriate enrolment of patients into RCTs.

**Methods:**

EMBASE, MEDLINE, Cochrane Database of Systematic Reviews, Cochrane Methodology Register, online abstract repositories, and conference websites were searched. Experts were contacted and bibliographies of retrieved papers hand-searched. The search cut-off date was 31 August 2009.

**Results:**

No primary publications were found. We identified one study in the grey literature (conference abstracts and presentations) reporting the results of an evaluation of the effectiveness of an intervention designed to prevent or avoid inappropriate enrolment of patients into an RCT. In the context of a multicentre trial, use of a dummy enrolment run-in phase was shown to reduce enrolment errors significantly (*P *< 0.001), from 16.1% during the run-in phase to < 1% after trial initiation.

**Conclusions:**

Our systematic search yielded only one technique or intervention shown to improve adherence to eligibility criteria during enrolment into RCTs. Given the potential harm involved in recruiting patients into a clinical trial in violation of key eligibility criteria, future research is needed to better inform those conducting clinical trials of how best to prevent enrolment errors

## Background

Within the context of a clinical trial, the primary purpose of defining clear, objective and precise study eligibility criteria is to facilitate the repeatable inclusion of patients who are thought most likely to benefit from the new drug or intervention under study, and to allow the exclusion of patients thought most likely to be harmed [1]. Inappropriate enrolment of patients in violation of key eligibility criteria may lead to excess avoidable harm to those patients, which may include serious adverse events or even death, attributable to the use of the study intervention in patients with important contraindications [2]. For the purpose of this review, we defined such an inappropriate enrolment as an enrolment error, and for the sake of clarity we defined the term 'eligibility criteria' to include both inclusion and exclusion criteria.

Authoritative sources recommend that if the proportion of ineligible patients recruited into a clinical trial exceeds 10%, the trial organisation should be considered to be 'generally poor' and may need 'tightening up' [1]. An overall estimate of enrolment error rates from clinical trials conducted in all disciplines of medicine is unavailable. In the discipline of intensive care medicine, enrolment error rates reported by multicentre Food and Drug Administration (FDA) licensing trials range from 9.4% (159/1690) [2] to 16.5% (77/464) [3]. Furthermore, evidence exists to suggest that enrolment errors may be preventable [2].

The highest risk of generating an enrolment error occurs when a new site recruits their first patient into a new trial. The first-patient enrolment error rate may be double the error rate for any subsequent patients [2]. Because site-specific enrolment errors have been shown to decrease as the number of patients enrolled at a site increases, the presence of a 'learning curve' has been proposed. As a site gains experience with the application of a particular study's eligibility criteria, they learn how to more appropriately apply each unique study inclusion and exclusion criterion, and are thus able to prevent or avoid enrolment errors [2]. Because the prevention or avoidance of enrolment errors may reduce avoidable harm, prevention or avoidance should take precedence over post-error detection [1].

The purpose of this review was to systematically identify studies evaluating the effectiveness of techniques or interventions designed to prevent or avoid enrolment errors during recruitment into randomised controlled trials (RCTs).

## Methods

### Primary literature search

To detect papers reporting the effectiveness of interventions designed to reduce enrolment errors in clinical trials, our primary search was conducted using MEDLINE http://www.PubMed.org, EMBASE http://www.EMBASE.com, the Cochrane Database of Systematic Reviews and the Cochrane Methodology Register http://www.mrw.interscience.wiley.com/cochrane/. Bibliographies of retrieved papers were also hand searched.

The primary search strategy was developed to identify any publication that reported protocol violations, protocol adherence or other related concepts. A complete list of these concept-related terms is presented in Table 1. In MEDLINE, concept-related terms were crossed with sensitive (broad) statements to identify clinical trials publications [4] and with the MeSH term 'clinical trials as topic'. An example of one of the main MEDLINE search strategies is provided in Additional file 1. In searches of other databases, all search strategies and terms developed for MEDLINE were mapped to database-specific indexing terms (for example, MeSH terms were mapped to EMTREE terms). EMTREE concept-related terms were crossed with sensitive (broad) EMTREE statements to identify publications related to clinical trials [5]. A complete list of terms and strategies developed for each database is available upon request from the authors.

**Table 1:** Search terms used in the primary literature search.

Databases	Specific search terms used
MEDLINE using Pubmed^a^; EMBASE^b^; Cochrane Database of Systematic Reviews	Protocol violation
	Recruitment violation
	Enrolment violation
	Recruitment error
	Protocol error
	Enrolment error
	Protocol adherence
	Protocol compliance
	Recruitment adherence

^a^Each of the terms were crossed (Boolean AND) with the Medline strategy described by Haynes *et al. *[4] for detecting RCTs. In addition, each was also crossed with the MeSH term 'clinical trials as topic'.

^b^Each of the terms were crossed (Boolean AND) with the EMBASE strategy described by Wong *et al. *[5] for detecting RCTs.

### Grey literature search

To identify the grey literature (for example, abstracts and presentations), we reviewed our own copies of conference booklets and searched for online abstract repositories. Conferences on the topic of clinical trials were identified by visiting recognized international society websites and by conducting a primary web search using the search engine Google http://www.google.com. These searches were conducted independently by two authors (EAS and GSD).

We also contacted recognised experts in the field. We defined 'recognised experts' as individuals with extensive experience in the design and conduct of clinical trials with a known interest in, or publications on, the topic of protocol violations. Expert consultation was conducted over several years, and took the form of informal discussions at conferences, management committee meetings and other venues, with the intent of learning how to reduce protocol violations.

### Search limits and date

The search was not limited by language or study type. The cut-off date was 31 August 2009.

### Study selection

All identified abstracts were independently reviewed by at least two authors (EAS and GSD), who were not blinded to publication source or abstract author list. Any abstract that either author believed was describing an intervention designed to reduce any form of protocol error or violation was retrieved in full text for detailed review. Non-English abstracts were translated.

An enrolment error was defined as the recruitment of a patient into a clinical trial in violation of an explicit, known eligibility criterion. Only papers reporting enrolment errors were eligible for further consideration. All techniques and interventions recommended to prevent or avoid enrolment errors were considered, and any form of evaluation of effectiveness was accepted.

Detailed, unblinded review of full text papers was independently conducted by all three authors (FS, EAS and GSD). Disagreements regarding paper inclusion were resolved by discussion and consensus. For each included paper, the following data items were independently extracted by two authors (FS, GSD): description of technique or intervention used to prevent/reduce enrolment errors, type of study undertaken to evaluate effectiveness of the technique or intervention in preventing or reducing enrolment errors, key design characteristics relevant to type of study, results of the evaluation in the context of specific outcomes reported, and description of the clinical population and the clinical treatment intervention type targeted to reduce enrolment errors (for example, medical discipline surgical intervention).

## Results

### Primary literature search

**Figure 1:**
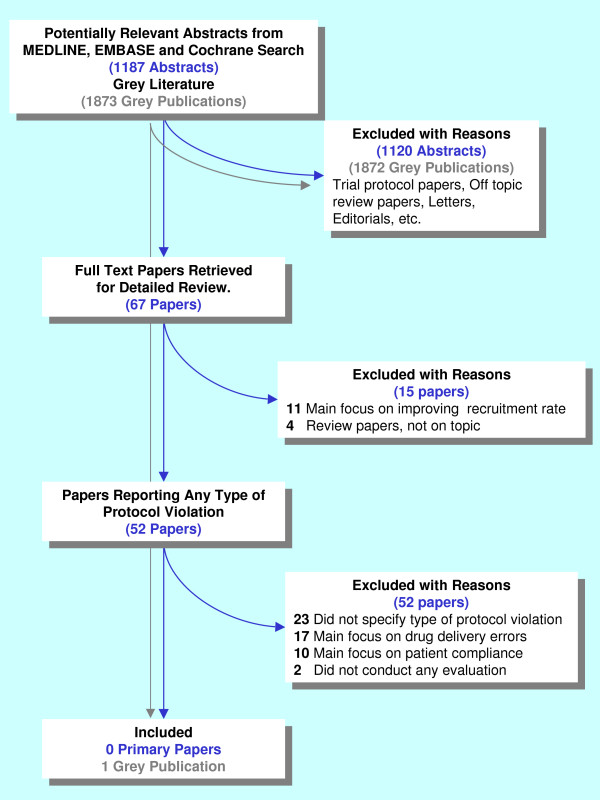
**The literature search study selection process**.

Search of MEDLINE, EMBASE, the Cochrane Database of Systematic Reviews and the Cochrane Methodology Register yielded 1187 unique, potentially relevant abstracts. On review, 67 full text papers were retrieved and reviewed in detail. No papers on the topic were identified by the primary search. Figure 1 provides a complete description of the selection process.

### Grey literature search

Recognised International society websites such as those of the Society for Clinical Trials [http://www.sctweb.org, accessed 26 October 2009], the International Society for Clinical Biostatistics [http://www.iscb.info, accessed 26 October 2009] and the Pharmaceutical Users Software Exchange [http://www.phuse.eu, accessed 26 October 2009] were found to be useful portal sites for identifying conferences on topic.

Although most conference websites published the titles of invited talks, and many academic conference websites provided access to the invited speakers' slides, no conference websites provided access to searchable abstract repositories. When available, titles of talks and abstracts were hand-searched. No talks or abstracts on topic were identified by the online search. One on-topic abstract was identified through contact with experts and review of conference booklets [6] (Figure 1). Complete agreement was reached on which studies should be included.

### Included studies

#### Dummy enrolment run-in phase

The results of this evaluation were published in abstract form only [6], and access to the complete content of the authors' slide presentation was provided [http://www.evidencebased.net/files/doig_runinphasever2.pdf, accessed 1 November 2009]. This evaluation was performed in the context of a multicentre RCT conducted in the intensive care units (ICUs) of 32 metropolitan and community hospitals throughout Australia and New Zealand (Australia and New Zealand Clinical Trials Registry, number 012605000704695).

The dummy enrolment run-in phase was undertaken before the formal live initiation of the study at each of the 32 participating ICUs, after site investigators and research coordinators attended 2-day educational study start-up meetings. During the dummy enrolment run-in phase, the sites screened patients for eligibility and applied the key inclusion and exclusion criteria to identify patients they believed to be eligible for the trial. De-identified demographic and eligibility information was submitted to the trial's clinical coordinating centre (CCC) using a secure, password-protected, encrypted study website. Patients in the run-in phase were not allocated to groups (treatment or control). Upon submission, the CCC reviewed the patient and provided timely feedback about their appropriateness for trial recruitment. After a site had submitted three consecutive truly eligible patients, the site was made 'live', and was able to begin recruiting and randomising patients into the trial.

During the dummy run-in phase, 32 sites submitted 199 potentially eligible patients for adjudication by the CCC. The median number of patients submitted per site was 4, (range 3 to 21). Thirteen sites progressed through the run-in phase without encountering enrolment errors. Two sites were required to submit 21 patients each before 3 consecutive truly eligible patients were identified at either site. The overall enrolment error rate during the dummy enrolment run-in phase was 16.1% (32/199).

As of June 2008, 409 patients had been formally recruited and randomised into the 'live' trial. The overall enrolment error rate during the actual conduct of the trial was 1% (4/409). Compared with the dummy enrolment run-in phase, enrolment error rates were significantly lower during the conduct of the actual trial (32/16 vs 4/409, *P *< 0.001, χ ^2 ^test).

## Discussion

We conducted a comprehensive and thorough search to identify studies evaluating the effectiveness of techniques or interventions designed to prevent or avoid enrolment errors during patient recruitment into RCTs. This included an attempt to search the grey literature (conference abstracts and presentations). No primary publications on this topic were found. We identified one study reported in the grey literature presenting the results of an evaluation of the effectiveness of an intervention (a dummy enrolment run-in phase) designed to prevent or avoid enrolment errors in a clinical trial. In the context of a multicentre clinical trial, use of such an intervention could significantly reduce enrolment errors; in this report, from 16.1% during the run-in phase to < 1% after trial initiation (*P *< 0.001).

### Background

The conduct of multicentre clinical trials is expensive, and the costs are increasing at an alarming rate. Accounting for all costs of development (including the costs of therapies that are not successful at the FDA phase I, II or III licensing stages, but not including marketing costs), the 1999 costs of successfully licensing one new drug or therapy under the FDA scheme have been estimated at US$500 million [7]. In 2009, discounting for inflation, these costs were estimated at more than US$1,000 million dollars [7]. Interestingly, over the same time period, the rate at which FDA phase III clinical trials failed to demonstrate expected benefits (negative trials) has risen from 20% in the 1990s to nearly 50% now. Excessive protocol violations may be one reason why trials fail to demonstrate expected benefits, even if benefits truly exist [2].

Excessive protocol violations may result in safety issues that can cause multicentre clinical trials to be stopped prematurely [8]. Data from multicentre FDA phase III licensing trials show that patients enrolled with protocol violations may experience harm, even death [2]. Furthermore, this harm may dilute treatment effects, leading to negative results or early trial cessation [9]. Examples of protocol violations include failure of the researchers to deliver the study intervention according to the study protocol, noncompliance of patient participants with the study protocol, issues relating to informed consent, and inappropriate enrolment of patients into the trial who did not meet the trial eligibility criteria. Of these various types of protocol violations, evidence suggests that enrolment in contradiction of key eligibility criteria (enrolment errors) can result in the greatest patient harm [2]. For example, if a protocol violation occurs because investigators fail to deliver any of the active study treatment, no direct patient harm is caused; however if a patient with a known contraindication to the study treatment is enrolled, the risk of harm is real.

Although it is recommended that study eligibility criteria should be clear, objective and precise [1], they are often complex and open to interpretation. In our own personal experiences, a CCC must dedicate considerable time during the early stages of a multicentre trial to ensure that interpretation is consistent between sites. Indeed, compelling evidence demonstrates that the risk of enrolment errors is highest early in a trial, and decreases as study sites gain experience in enrolling patients [2,9]. This apparent learning curve, combined with the possibility of harm from inappropriate enrolment, begs the question: how can we get to the flat end of the learning curve faster?

### The dummy enrolment run-in phase

Medical simulation exercises, such as performing cardiopulmonary resuscitation on a mannequin, can create a realistic learning environment without patient risk. Simulations have been shown to change practice, increase compliance with guidelines, and improve team dynamics [10]. Like a simulation exercise, a dummy enrolment run-in phase in an RCT replicates the screening and patient identification process in a protected and interactive learning environment, without patient risk.

The dummy enrolment run-in phase is conducted after the provision of education at a study start-up meeting but before an enrolment website is made 'live'. During the dummy enrolment run-in phase, sites gain practical experience applying the new study eligibility criteria by attempting to correctly identify patients they believe to be eligible for the trial and submitting these patients to the trial's CCC for adjudication on appropriateness. Because these patients are not formally enrolled or randomised, risk of harm is avoided. The dummy enrolment run-in phase allows participating sites to learn, by direct first-hand experience, how to apply and interpret novel inclusion and exclusion criteria [11].

If a site submits a patient that is inappropriate to enrol, the CCC is provided with an opportunity to provide immediate nonpunitive positive feedback and education on that particular patient. The dummy enrolment run-in phase also allows the CCC to identify sites that may have major problems with trial execution. These sites can be targeted and provided with more detailed multifaceted educational strategies, including educational outreach visits and formal one to one academic detailing [12,13], before making the study live. Furthermore, the dummy enrolment run-in phase allows the CCC to learn as well.

The CCC can use the dummy enrolment run-in phase to identify inclusion or exclusion criteria that are too complex, open to different interpretation or poorly worded. These problematic criteria can be addressed by providing additional information to all sites using a 'frequently asked questions' (FAQ) publication. The CCC may also elect to rewrite problematic criteria or to change the focus or content of future education on the topic. The dummy enrolment run-in phase provides the opportunity to refine CCC processes and address eligibility criteria issues before the trial goes live.

The conduct of a dummy enrolment run-in phase increases the overall duration, and therefore the costs, of a clinical trial. The ideal duration of the run-in phase must therefore be balanced against these costs. The publications by Macias *et al*. and Laterre *et al*. both present evidence of a 'learning curve', whereby more protocol violations occur early during a clinical trial, and suggest that error rates are minimised after a site recruits at least four patients [2,9]. The presence of learning curves, describing how complications are reduced as experience increases, have been reported for many laparoscopic and other surgical procedures [14-16]. It is likely that the ideal duration of a dummy enrolment run-in phase varies based on the complexity of the eligibility criteria and on other trial factors. Publications of enrolment errors and other protocol violations by patient recruitment numbers should be encouraged to assist those conducting trials in determining the appropriate duration of a dummy enrolment run-in for their given speciality, discipline or type of trial.

### Strengths and limitations

The primary literature search, conducted using PubMed, EMBASE and Cochrane database, was designed to be 'sensitive' to the presence of articles on the topic of interest. We are reasonably certain that our negative search results are reliable. Because we acknowledged that it was possible the primary literature search would yield few results, we believed it was important to search the grey literature as well, and, for the purposes of this study, focused this 'grey literature search towards identifying conference abstracts and presentations [17].

Hand-searching for conference abstracts is known to be time-consuming, expensive and difficult [18]. Our grey literature search for online abstract repositories was unrewarding. Because conference websites are not indexed using standardised terms, it is likely that our search missed important meetings. Online abstract repositories were uncommon, even though the technology for providing access to abstracts in a searchable format is readily available. For example, posting the abstract to the conference website in a portable document format (PDF) that is indexed by Google Scholar (http://scholar.google.com.au, accessed 26 October 2009) would effectively create an online searchable repository. We strongly recommend that academic conferences publish accepted abstracts on their conference website in a format that allows indexing and thus searching, by search engines such as Google Scholar.

## Conclusions

We undertook a comprehensive literature search with the express purpose of identifying techniques or interventions demonstrated to avoid or prevent enrolment errors during recruitment into RCTs. No primary publications on this topic were found. A search of the grey literature revealed one abstract evaluating the effect of a dummy enrolment run-in phase on preventing enrolment errors in a multicentre RCT, which was found to significantly prevent enrolment errors after trial initiation.

Given the potential for harm when a patient is inappropriately enrolled into a clinical trial, the lack of research in this field is concerning. Further research is needed to identify and validate other techniques and interventions that can prevent or avoid enrolment errors. Research is also needed to identify factors, such as lack of peer review, number of participating sites, and overall trial size or budget, which may predispose a trial to excessive enrolment errors. As an initial step, we recommend that those conducting clinical trials begin reporting protocol errors by type in the primary results publications of clinical trials.

## Competing interests

The authors declare that they have no competing interests.

## Authors' contributions

FS, EAS and GSD conceived and designed the study, interpreted the results of the literature search, and drafted the manuscript.

## Supplementary Material

Additional file 1 **Appendix 1**. Example of a main search strategy, in MEDLINE syntax. Click here for file
