# Supplementary material for: A systematic review of techniques and interventions for improving adherence to inclusion and exclusion criteria during enrolment into randomised controlled trials
Source: Trials. 2010 Feb 23;11:17. doi: 10.1186/1745-6215-11-17 (PMC2838880; doi:10.1186/1745-6215-11-17)
Supplement: Additional file 1 — Appendix 1. Example of a main search strategy, in MEDLINE syntax. [file 1745-6215-11-17-S1.DOC]

**Appendix 1**: Example of a Main Search Strategy, in MEDLINE syntax.

| Database | Search Strategy, in MEDLINE syntax |
| --- | --- |
| MEDLINE using PubMed | (recruitment[All Fields] AND adherence[All Fields]) AND (((clinical[Title/Abstract] AND trial[Title/Abstract]) OR "clinical trials as topic"[MeSH Terms] OR clinical trial[Publication Type] OR (random[Title/Abstract] OR random/aleatory[Title/Abstract] OR random/and[Title/Abstract] OR random/arm[Title/Abstract] OR random/basal[Title/Abstract] OR random/blind[Title/Abstract] OR random/blocked[Title/Abstract] OR random/cdp[Title/Abstract] OR random/clustered[Title/Abstract] OR random/combinatorial[Title/Abstract] OR random/double[Title/Abstract] OR random/ethanol[Title/Abstract] OR random/half[Title/Abstract] OR random/linkage[Title/Abstract] OR random/mixed[Title/Abstract] OR random/nonrandom[Title/Abstract] OR random/random[Title/Abstract] OR random/representative[Title/Abstract] OR random/systematic[Title/Abstract] OR random/turns[Title/Abstract] OR random'[Title/Abstract] OR random1[Title/Abstract] OR randomally[Title/Abstract] OR randomaly[Title/Abstract] OR randomamplified[Title/Abstract] OR randoman[Title/Abstract] OR randomand[Title/Abstract] OR randomate[Title/Abstract] OR randombred[Title/Abstract] OR randombreds[Title/Abstract] OR randomdigit[Title/Abstract] OR randomdot[Title/Abstract] OR randome[Title/Abstract] OR randomed[Title/Abstract] OR randomeffects[Title/Abstract] OR randomely[Title/Abstract] OR randomer[Title/Abstract] OR randomezed[Title/Abstract] OR randomforest[Title/Abstract] OR randomi[Title/Abstract] OR randomiazed[Title/Abstract] OR randomic[Title/Abstract] OR randomically[Title/Abstract] OR randomicaly[Title/Abstract] OR randomiced[Title/Abstract] OR randomicity[Title/Abstract] OR randomied[Title/Abstract] OR randomifzed[Title/Abstract] OR randomil[Title/Abstract] OR randomily[Title/Abstract] OR randomined[Title/Abstract] OR randomingly[Title/Abstract] OR randominzed[Title/Abstract] OR randomirrespective[Title/Abstract] OR randomis[Title/Abstract] OR randomisation[Title/Abstract] OR randomisation'[Title/Abstract] OR randomisations[Title/Abstract] OR randomisationsecondary[Title/Abstract] OR randomise[Title/Abstract] OR randomised[Title/Abstract] OR randomised/controlled[Title/Abstract] OR randomised/crossover[Title/Abstract] OR randomised'[Title/Abstract] OR randomisee[Title/Abstract] OR randomisely[Title/Abstract] OR randomises[Title/Abstract] OR randomisiert[Title/Abstract] OR randomisierte[Title/Abstract] OR randomising[Title/Abstract] OR randomisly[Title/Abstract] OR randomiz[Title/Abstract] OR randomizable[Title/Abstract] OR randomizadely[Title/Abstract] OR randomizado[Title/Abstract] OR randomizaion[Title/Abstract] OR randomization[Title/Abstract] OR randomization/baseline[Title/Abstract] OR randomization/blinding[Title/Abstract] OR randomization/matching[Title/Abstract] OR randomization/nonspecificity[Title/Abstract] OR randomization/permutation[Title/Abstract] OR randomization/registration[Title/Abstract] OR  randomization/selection[Title/Abstract] OR randomization/stabilization[Title/Abstract] OR  randomization/start[Title/Abstract] OR randomization'[Title/Abstract] OR  randomizations[Title/Abstract] OR randomizd[Title/Abstract] OR  randomize[Title/Abstract] OR randomized[Title/Abstract] OR  randomized/15[Title/Abstract] OR randomized/455[Title/Abstract] OR  randomized/58[Title/Abstract] OR randomized/blind[Title/Abstract] OR  randomized/blinded[Title/Abstract] OR randomized/controlled[Title/Abstract] OR  randomized/counterbalanced[Title/Abstract] OR randomized/nonrandomized[Title/Abstract] OR randomized/organized[Title/Abstract] OR randomized/prospective[Title/Abstract] OR randomized/quasirandomized[Title/Abstract] OR  randomized/registry[Title/Abstract] OR randomized/screened[Title/Abstract] OR  randomized/stratified[Title/Abstract] OR randomized'[Title/Abstract] OR  randomized150[Title/Abstract] OR randomizedcounterbalanced[Title/Abstract] OR  randomizedduring[Title/Abstract] OR randomizedly[Title/Abstract] OR  randomizedphase[Title/Abstract] OR randomizedtrial[Title/Abstract] OR  randomizely[Title/Abstract] OR randomizer[Title/Abstract] OR  randomizer'[Title/Abstract] OR randomizes[Title/Abstract] OR  randomizied[Title/Abstract] OR randomizing[Title/Abstract] OR  randomizing'[Title/Abstract] OR randomizzati[Title/Abstract] OR  randoml[Title/Abstract] OR randomlike[Title/Abstract] OR  randomlly[Title/Abstract] OR randomly[Title/Abstract] OR  randomly/linearly[Title/Abstract] OR randomly'[Title/Abstract] OR  randomlyassigned[Title/Abstract] OR randomlyselected[Title/Abstract] OR  randommess[Title/Abstract] OR randommethacrylic[Title/Abstract] OR  randommized[Title/Abstract] OR randommobility[Title/Abstract] OR  randomnes[Title/Abstract] OR randomness[Title/Abstract] OR  randomness'[Title/Abstract] OR randomnesses[Title/Abstract] OR  randomnicity[Title/Abstract] OR randomnly[Title/Abstract] OR  randomomized[Title/Abstract] OR randoms[Title/Abstract] OR  randomsample[Title/Abstract] OR randomsed[Title/Abstract] OR  randomsource[Title/Abstract] OR randomwalksat[Title/Abstract] OR  randomy[Title/Abstract] OR randomyl[Title/Abstract] OR  randomyly[Title/Abstract] OR randomzed[Title/Abstract] OR  randomzied[Title/Abstract] OR randomzing[Title/Abstract]) OR "random  allocation"[MeSH Terms] OR "therapeutic use"[Subheading]) OR ("clinical  trials as topic"[All Fields])) |
